# Threat Memory Reminder Under Matrix Metalloproteinase 9 Inhibitor Doxycycline Globally Reduces Subsequent Memory Plasticity

**DOI:** 10.1523/JNEUROSCI.1285-19.2019

**Published:** 2019-11-20

**Authors:** Dominik R. Bach, Monika Näf, Markus Deutschmann, Shiva K. Tyagarajan, Boris B. Quednow

**Affiliations:** ^1^Computational Psychiatry Research, Department of Psychiatry, Psychotherapy and Psychosomatics, University of Zurich, 8032 Zurich, Switzerland,; ^2^Neuroscience Centre Zurich, University of Zurich, 8057 Zurich, Switzerland,; ^3^Wellcome Centre for Human Neuroimaging and Max Planck UCL Centre for Computational Psychiatry and Ageing Research, University College London, London WC1N 3BG, United Kingdom,; ^4^Experimental and Clinical Pharmacopsychology, Department of Psychiatry, Psychotherapy and Psychosomatics, University of Zurich, 8032 Zurich, Switzerland, and; ^5^Institute of Pharmacology and Toxicology, University of Zurich, 8057 Zurich, Switzerland

**Keywords:** fear-potentiated startle, matrix metalloproteinase, psychophysiological modeling, reconsolidation, threat conditioning, trauma-related disorder

## Abstract

Associative memory can be rendered malleable by a reminder. Blocking the ensuing reconsolidation process is suggested as a therapeutic target for unwanted aversive memories. Matrix metalloproteinase-9 (MMP-9) is required for structural synapse remodeling involved in memory consolidation. Inhibiting MMP-9 with doxycycline is suggested to attenuate human threat conditioning. Here, we investigated whether MMP-9 inhibition also interferes with threat memory reconsolidation. Male and female human participants (*N* = 78) learned the association between two visual conditioned stimuli (CS^+^) and a 50% chance of an unconditioned nociceptive stimulus (US), and between CS^−^ and the absence of US. On day 7, one CS^+^ was reminded without reinforcement 3.5 h after ingesting either 200 mg of doxycycline or placebo. On day 14, retention of CS memory was assessed under extinction by fear-potentiated startle. Contrary to our expectations, we observed a greater CS^+^/CS^−^ difference in participants who were reminded under doxycycline compared with placebo. Participants who were reminded under placebo showed extinction learning during the retention test, which was not observed in the doxycycline group. There was no difference between the reminded and the nonreminded CS^+^ in either group. In contrast, during relearning after the retention test, the CS^+^/CS^−^ difference was more pronounced in the placebo group than in the doxycycline group. To summarize, a single dose of doxycycline before threat memory reminder appeared to have no specific impact on reconsolidation, but to globally impair extinction learning, and threat relearning, beyond drug clearance.

**SIGNIFICANCE STATEMENT** Matrix metalloproteinase-9 inhibition appears to attenuate memory consolidation. It could also be a target for blocking reconsolidation. Here, we test this hypothesis in human threat conditioning. We find that doxycycline has no specific impact on a reminded cue, but confers a global reduction in extinction learning and threat learning beyond the clearance of the drug. This may point toward a more long-lasting impact of doxycycline treatment on memory plasticity.

## Introduction

Recall can render associative memory malleable under suitable conditions (Nader et al., 2000). Such labilized memory is thought to spontaneously stabilize in a reconsolidation process. This has been demonstrated by disrupting reconsolidation with local protein synthesis inhibition, which makes conditioned responding disappear (Nader et al., 2000). While extinction training also attenuates conditioned responding, the initial threat memory can reemerge after passage of time, in a different context, or after nonpredictable unconditioned stimulus (US) presentations (Dunsmoor et al., 2015). This is not (or less so) the case for reconsolidation blockade, which thus appears to lastingly modify memory (Duvarci and Nader, 2004; Lin et al., 2006). Thus, reconsolidation blockade could be a potentially powerful principle for clinical treatment of unwanted aversive memories, such as the recollection of psychological trauma (Kindt, 2018).

Because systemically administering protein synthesis inhibitors is not feasible, previous attempts to translate this approach to humans have capitalized on behavioral procedures such as reminder/extinction combination (Monfils et al., 2009; Schiller et al., 2010) or neurotransmitter-based mechanisms such as norepinephrine antagonists (Debiec and Ledoux, 2004; Kindt et al., 2009; Brunet et al., 2018). However, it may also be possible to interfere more directly with intrasynaptic signaling pathways to achieve this goal. Conceptually, reconsolidation could be a way of integrating new information into existing memory, and is therefore often thought to be similar to consolidation (McKenzie and Eichenbaum, 2011). Indeed, many (although not all) molecular and cellular features of consolidation and reconsolidation are shared (Besnard et al., 2012). Here, we focus on MMP-9, which is a key molecule in the consolidation pathway (Huntley, 2012) and can be targeted with human-approved drugs (Bach et al., 2018a).

MMP-9 forms part of a signaling cascade that leads to the persistent structural changes in the synaptic configuration that underlie long-term potentiation (LTP) (Huntley, 2012). MMP inhibition or knock-out disrupts LTP in acute slices (Nagy et al., 2006; Meighan et al., 2007; Okulski et al., 2007; Wang et al., 2008; Gorkiewicz et al., 2015), while activated MMP-9 induces LTP (Nagy et al., 2006; Wang et al., 2008). *In vivo*, MMP inhibition reduces spatial and contextual learning in nonhuman animals (Nagy et al., 2007; Knapska et al., 2013). Translating these findings to humans is afforded by the antibiotic doxycycline, a broad-spectrum MMP inhibitor (Hanemaaijer et al., 1998) that crosses the blood-brain barrier (Mento et al., 1969; Dotevall and Hagberg, 1989; Karlsson et al., 1996; Lucchetti et al., 2019). Using a standard delay discriminative threat conditioning protocol (also termed fear conditioning; LeDoux, 2014), we have previously shown in humans that a single dose of 200 mg of doxycycline, administered orally ∼210 min before a multiple-trial Pavlovian discriminative threat learning procedure, reduced retention of that memory on day 7 by ∼60% (Bach et al., 2018a). This suggests that doxycycline interferes with acquisition and/or synaptic consolidation, consistent with an impact on LTP. If the synaptic mechanisms underlying consolidation and reconsolidation are to some extent similar, this raises a possibility that doxycycline may also interfere with synaptic reconsolidation. A rodent study yielded ambiguous evidence for this possibility: reconsolidation was disrupted after reminder under MMP inhibition in animals that had undergone four-trial threat conditioning (Brown et al., 2009). In the same report, however, there was no impact of MMP inhibition on synaptic consolidation in one-trial Pavlovian threat conditioning (Brown et al., 2009). Here, we sought to demonstrate an impact of doxycycline on threat memory reconsolidation in humans.

## Materials and Methods

### 

#### Participants

**Figure 1.**
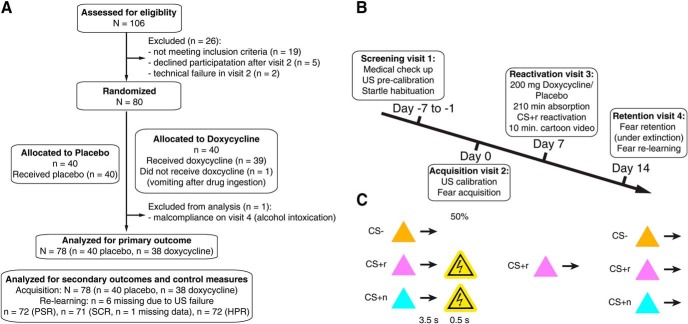
Study design. ***A***, CONSORT flowchart. ***B***, Study procedures. ***C***, Three CS were trained on day 0, of which two were reinforced with 50% rate. On day +7, one of these, CSr^+^, was reminded without reinforcement. On day +14, threat memory retention was tested under extinction, i.e., without reinforcement. Afterward, US was presented again in a relearning test (not shown in this figure).

Participants were recruited from the general population (*N* = 80; 40 per group; 20 female per group). One participant did not complete reminder visit 3 due to vomiting immediately after ingesting the drug. One further participant was excluded from analysis due to suspected alcohol consumption before retention visit 4. Re-including this participant into the analysis did not change the pattern of results. The reported final sample therefore comprised 78 individuals, 40 in the placebo group and 38 in the doxcycline group (Fig. 1*A*). The groups did not differ in age, gender, US intensity depression, state anxiety, or trait anxiety (Table 1). Differences in accuracy during acquisition were modeled as covariates. All participants were screened for health conditions by a physician during visit 1 (see Bach et al., 2018a for in- and exclusion criteria).

**Table 1.** Demographic and performance characteristics of the final analyzed sample

Sex	Placebo		Doxycycline		
	20 male	20 female	20 male	18 female	
	Mean	SD	Mean	SD	*p^b^*
Age	24.4	4.84	25.3	4.97	0.41
STAI X1	34.8	6.89	35.70	5.68	0.52
STAI X2	37.1	6.40	38.4	5.41	0.31
BDI	3.74	4.35	3.19	3.27	0.82
US current (mA)	3.87	1.06	3.97	1.54	0.75
US habituation during acquisition (rating difference)	−5.21	13.6	−6.22	15.8	0.86
US habituation end of acquisition, end of relearning (rating difference)*^a^*	9.00	15.0	−7.2	14.6	0.62
Accuracy acquisition	0.97	0.07	0.99	0.02	0.11
Accuracy reminder	0.93	0.27	0.87	0.34	0.42
Accuracy retention/relearning	0.99	0.02	0.99	0.02	0.96
Performance acquisition (response rate)	0.99	0.01	1.00	0.01	0.12
Performance reminder (response rate)	0.97	0.16	0.97	0.16	0.97
Performance retention/relearning (response rate)	1.00	0.00	1.00	0.01	0.36
RT acquisition (ms)	953	214	996	226	0.44
RT reminder (ms)	1186	607	1103	447	0.51
RT retention/relearning (ms)	927	219	948	228	0.69
Number of response training blocks required	1.55	1.08	1.44	0.55	0.60

*^a^*Six participants were not included into analysis of the relearning session (see Materials and Methods, “Participants”).

*^b^p*-value from a two-sample, two-tailed *t* test comparing the two groups.

STAI, State-Trait Anxiety Inventory; X1, state anxiety; X2, trait anxiety; BDI, Beck Depression Inventory.

US habituation indicates an average pain rating (0–100) difference. Accuracy indicates correct responses/total trials in incidental task. Performance indicates total responses/total trials in incidental task.

The study was conducted in accord with the Declaration of Helsinki and approved by the governmental research ethics committee (Kantonale Ethikkomission Zurich, KEK-ZH 2014–0669) and the Swiss Agency for Therapeutic Products (Swissmedic, 2015DR1136). All participants gave written informed consent using a form approved by the ethics committee. The study was preregistered at the primary ISRCTN registry (ISRCTN66987216) and at the Swiss Federal Complementary Database (KOFAM; SNCTP000001439).

#### Power analysis

Power analysis was based on a pilot study with the same setup (Khemka et al., 2017b; see Bach et al., 2018a for details). A sample size of *N* = 74 was required to achieve 80% power to detect at least 50% reduction in threat memory at an α rate of 0.05. We recruited *N* = 80 participants to allow for attrition.

#### Study medication

##### Drug production and dosage.

The study medication was doxycycline, brand name Vibramycin (Pfizer). A GMP-licensed pharmacy (Kantonsapotheke Zürich) manufactured, blinded, and randomized the study medication separately for males and females; mannitol was used as placebo. Randomization code was broken after the last participant completed the study, and after all data were checked for consistency. The study dose of 200 mg is the smallest antibiotic dose recommended by the manufacturer and the same dose that yielded a 60% reduction in threat memory consolidation in a previous report (Bach et al., 2018a).

##### Timing of the reminder.

In healthy individuals, plasma *t*_max_ of doxycycline preparations is on the order of ∼2 h, although not reported in humans for the galenic formulation used here (Gschwend et al., 2007). Similarly, in individuals treated for neuroborreliosis, plasma *t*_max_ on treatment day 13 was between drug measurements taken at 0 and 2 h for most individuals, and between measurements taken at 2 and 4 h for the remaining ones (Karlsson et al., 1996). Doxycycline crosses the blood–brain barrier and is used for treatment of Lyme disease. In patients treated for this condition, doxycycline was detectable in CSF 2–3 h after ingestion on treatment days 5–8 (Dotevall and Hagberg, 1989), and 4 h after oral ingestion on treatment day 13 (Karlsson et al., 1996); both studies report only one CSF measurement. In patients with schizophrenia, doxycycline was detectable in CSF 4 h after ingestion on treatment day 1 (Mento et al., 1969). In mice, repeated measurement of CSF levels revealed a CSF *t*_max_ of 4 or 6 h after intraperitoneal treatment, depending on the dose, with very little change between 4 and 6 h (Lucchetti et al., 2019). In a previous study, we had started threat memory acquisition after ∼3.5 h (Bach et al., 2018a). Here, we scheduled the memory reminder after 3.5 h for consistency.

##### Timing of the retention test.

Doxycycline's half-life is ∼16 h according to the manufacturer's information; such the drug was cleared by >99.9% at the retention test 7 d after ingestion.

#### Experimental procedures

##### Screening visit 1 (day −7 to day −1).

The study procedure is summarized in Figure 1*B*. On visit 1, we determined US intensity and tolerance to startle sounds, and performed medical examination to check exclusion criteria (Bach et al., 2018a).

##### Acquisition visit 2 (day 0).

Acquisition visit 2 took part between 08.00 and 15.30. Participants filled in the German translations of the State-Trait Anxiety Inventory (state: X1, trait: X2) (Laux et al., 1981) and Beck's Depression Inventory (Hautzinger et al., 1994), followed by the threat learning protocol. First, we recalibrated US intensity using the same random procedure as on screening visit 1. Participants then trained the color/response key-mapping in blocks of six balanced CS until they pressed the correct key in 5 of 6 trials in one block (see Table 1 for the average number of training blocks required). This was followed by a standard discriminant delay threat conditioning paradigm with 45 trials [15 CS^−^, 15 CS^+^ that is reminded on day +7 (CSr^+^), 15 CSn^+^ that is not reminded) in 1 block (Fig. 1*C*). Both CS^+^ coterminated with an electric stimulation as aversive US (see “Stimuli and recordings” section) in 50% of trials. Trial sequence was randomly balanced for each participant, with the restriction that the first trial of each phase was always a reinforced CS^+^, the first six trials of each phase included each CS exactly twice, and that there could not be >5 instances of the same CS and 4 instances of US, or US omission, in a row. As an incidental task, participants were instructed to press one of three cursor keys on a standard keypad to indicate CS color. We identified two outlier participants in the acquisition session: one (later treated with doxycycline) required an unusually high number of 7 training blocks (maximum for the rest of the sample: 3) and one (later treated with placebo) had an usually low accuracy of 56% in the incidental task. We conservatively retained these in the analysis but note that results of the primary analysis did not change if they were excluded.

##### Reminder visit 3 (day +7).

This visit took place between 08.00 and 17.00, with the reminder procedure finished before 16.00. Participants were verbally screened for health issues and ingested the study medication. During a 210 min absorption interval, they were kept under surveillance of study staff. They were then attached to all electrodes, including the US electrode in the same location as on visit 2. Participants were instructed that they might receive US, but that CS/US contingency was determined by the computer and unknown to the study assistant. They saw one reminder CSr^+^ without reinforcement. This procedure would induce a learning-theoretic prediction error, which has been suggested crucial to engage reconsolidation (Sevenster et al., 2013). The use of a single reminder trial in cue conditioning is in line with previous human work (Kindt et al., 2009; Schiller et al., 2010) and has been suggested suitable for engaging molecular reconsolidation (as opposed to extinction) processes in rats (Merlo et al., 2014). The timing of the reminder session, 7 d after acquisition, was chosen to facilitate participant scheduling. We note that reconsolidation blockade of 1 week- and even 3 week-old memories has been demonstrated in mice (Suzuki et al., 2004). After the reminder, all electrodes were removed, and participants watched a preselected 10 min cartoon movie episode with subtitles and without audio. This procedure is in line with previous human work (Schiller et al., 2010), and was chosen to bring cognitive effort immediately after the reminder under experimental control. This was followed by a 60 min neuropsychological assessment to investigate the impact of doxycycline on other cognitive functions, which will be reported elsewhere.

##### Retention visit 3 (day +14).

Participants were attached to all electrodes, including the US electrode in the same location as on visit 2. They were then instructed that they might receive US, but that CS/US contingency was determined by the computer and unknown to the study assistant. They saw 45 CS (15 CS^−^, 15 CSr^+^, CSn^+^) in randomly balanced order, and heard a startle probe 3.5 s after onset of all CS, but never received a US. Note that the motoric startle response makes psychophysiological data other than startle eye-blink responses from this session unusable. Immediately afterward, we measured relearning over 90 trials by coterminating 50% of CS^+^ with a US, without startle sounds. US delivery was not tested before relearning, to avoid reinstatement. Although US electrode location was controlled by measuring its distance from palpable carpal bones, minute differences in attachment can lead to diminished US perception. Seven participants (5 doxycycline, 2 placebo; Fisher's exact test, *p* = 0.26) showed no unconditioned SCR to the shock, including three participants who reported in the final US intensity assessment that they did not feel any US during relearning at all. One of these seven participants was already excluded due to suspected alcohol consumption; the other six were excluded for analysis of psychophysiological data in this session only. The first CS^+^ in this session was always reinforced, such that the first data point available for each CS^+^ was recorded after the first US.

#### Stimuli and recordings

##### Conditioned stimuli (CS).

CS were isoluminant colored triangles presented for 4 s, while the screen was gray during the intertrial interval, randomly determined to be 7 s, 9 s, or 11 s. CS colors were (RGB values) orange (255, 176, 0), violet (255, 125, 255) and turquoise (0, 255, 255), while the background was gray (179, 179, 179) with a white fixation cross.

##### US.

The US was a 500 ms train of 250 electrical square pulses with an individual pulse duration of 0.2 ms, delivered on participants' dominant forearm through a pin-cathode/ring-anode configuration with a constant current stimulator (DS7A; Digitimer). The current was set such that perceived shock intensity was ∼90% of the pain threshold. We initially (visit 1) estimated the pain threshold during two phases. First, the intensity was increased from being unperceivable to a painful level. This was set as upper limit for all following perception tests, in which participants were asked to rate the perceived intensity of 14 stimuli with different currents, which participants rated on a scale from 0 (not perceived) to 10 (clearly painful). Ratings were interpolated to estimate the current that the participant would have been rated as 90%. This current was then individually adjusted to yield a clearly discomforting but not painful stimulus. US electrode positioning across visits was ensured by recording distance from the (palpable) carpal bones. On acquisition visit 2, US perception was controlled with 14 stimuli of random intensity before threat memory acquisition. Stimulation strength was modified if necessary to yield a clearly discomforting but not painful stimulus. On reminder visit 3, US electrodes were attached and the stimulator was turned on, but no US were delivered. On retention visit 4, US electrodes were attached and no US were delivered before the tasks started. In both acquisition visit 2 and retention visit 4, pain perception was controlled after the task using 14 random stimuli. For part of the sample, different random stimuli were used in different assessments. For those participants that received the same random stimuli across two subsequent assessments, perceived US intensity decreased from beginning to end of acquisition visit 2 (*t*_(43)_ = −2.6, *p* = 0.012) and from end of acquisition visit 2 to end of relearning on visit 4 (*t*_(67)_ = −4.5, *p* < 0.001; excluding 6 participants who did not show a SCR to the US on visit 4) with no difference between placebo and drug group (Table 1).

##### Startle probes.

In accordance with current recommendations (Blumenthal et al., 2005) and our own previous work (Khemka et al., 2017b), white noise bursts (loudness: 102 dB, duration: 40 ms, measured rise and fall time: < 2 ms, sampling frequency 44.1 kHz), were used as startle probes and delivered via headphones (HD 201; Sennheiser), using the PC's inbuilt sound card (Realtek high definition audio) and an external sound amplifier (K4102, Velleman, Belgium). Sound volume was determined offline using a white noise sound of 2 s duration and a sound level meter (SL-200; Voltcraft). Sound onset was controlled by recording the output of the sound card together with EMG, and all analyses relate to the measured startle sound onset.

##### Outcome measures.

Preregistered primary outcome measure was startle potentiation over the entire retention test, measured as startle eye blink response (SEBR) in the same way as in a previous report (Bach et al., 2018a). There were no missing data in the primary outcome. Preregistered secondary outcome measures were skin conductance responses (SCRs) and heart period responses (HPRs, i.e., conditioned bradycardia) during acquisition and relearning. We also recorded and analyzed pupil size because of its high fidelity (Korn et al., 2017) and because we had, after finalizing the preregistration, demonstrated that pupil size responses (PSRs) may be more closely related to US prediction than SCR (Tzovara et al., 2018).

##### Psychophysiological recordings.

The experiment took place in a dark, soundproof chamber. Participants placed their head on a chin rest at a distance of 70 cm from the monitor (Dell P2012H, 20-inch set to an aspect ratio of 5:4, 60 Hz refresh rate). SEBR were recorded using electromyogram from the orbicularis oculi muscle of participants' right eye and two 4 mm Ag/AgCl cup electrodes filled with high-conductance gel. One of them was placed 10 mm below the lower eyelid in line with the pupil in forward gaze and the other on the external canthus at a distance of 10 mm from the first (Blumenthal et al., 2005). Electromyogram was amplified with a Colbourn isolated bioamplifier (V75-11; Colbourn Instruments). Skin conductance was recorded from the thenar/hypothenar of participants' left hand, using 8 mm Ag/AgCl cup electrodes (EL258; Biopac Systems) and 0.5% NaCl gel (GEL101; Biopac) (Hygge and Hugdahl, 1985). Skin conductance signal was amplified with an SCR coupler/amplifier (V71-23; Coulbourn Instruments). All data were digitised at 1000 Hz using a DI-149 A/D card (Dataq Instruments, Akron, OH, US), and recorded with Windaq (Dataq Instruments) software. We recorded pupil area and gaze direction for both eyes with an EyeLink 1000 System (SR Research) situated 47 cm away from the participant's eyes. The sampling rate was 500 Hz. To calibrate gaze direction, we used the 9-point protocol implemented in the EyeLink 1000 software.

#### Psychophysiological modeling

For psychophysiological analysis, we used a MATLAB toolbox for psychophysiological modeling, PsPM (version 4.0.2 r575, pspm.sourceforge.net) (Bach and Friston, 2013; Bach et al., 2018b).

##### SEBR.

Electromyogram processing was performed in the same way as in a previous report (Bach et al., 2018a), using the most sensitive method from a previous methodological comparison in the same setup (Khemka et al., 2017b). We bandpass filtered the electromyogram signal with a fourth order Butterworth band-pass filter (50–470 Hz), and applied a notch filter to remove 50 Hz harmonics. Filtered electromyogram data were rectified and smoothed with a 3 ms (53.05 Hz) fourth order Butterworth low pass filter. We then inverted a psychophysiological model that quantifies, for each trial, amplitude of the SEBR by linear regression onto a canonical SEBR with variable onset (Khemka et al., 2017b). Recorded sound output was used as event marker. Differences in electrode impedance and muscle anatomy will result in a multiplicative scaling of the true SEBR. We thus normalized data by dividing each participant's single-trial SEBR estimates through the mean SEBR in CS^−^ trials in the same way as in our previous report (Bach et al., 2018a).

##### PSR.

Eye blinks and saccades were detected by the online parsing algorithm of the eye tracker and excluded as missing data. Periods during which gaze direction was outside a box with 5° visual angle around the screen center were excluded as well. The pupil with fewer missing data points was used for subsequent analysis. Missing data points were linearly interpolated for filtering and ignored during model inversion. A trial was excluded if there were <50% available data points during the 10 s following CS onset. This procedure excluded, across all participants 40 trials (1.1%) from acquisition, and 72 (0.4%) from re-relearning. No participant had >35% missing trials in any session. To estimate the anticipatory pupil response, we used a single-trial general linear convolution model (GLM) after down sampling the data to 250 Hz (Korn et al., 2017).

##### SCR.

SCR data were visually inspected by a rater blind to placebo/doxycycline condition, and artifact periods (temporary electrode detachment or signal clipping) were excluded. Artifact periods shorter than 2 s were linearly interpolated for filtering and ignored for model inversion. If longer artifact periods fell into a trial, then this trial was excluded. No SCR data were available for one participant during relearning (placebo) due to electrode detachment. For the acquisition session, we further removed (across participants) 2 trials (0.05%). SCR data were then filtered with a first order bidirectional bandpass Butterworth filter (cutoff frequencies: 0.0159–5 Hz, using interpolation for artifact periods), and down-sampled to 10 Hz. Resulting traces were analyzed by nonlinear inversion of a PsPM that describes the anticipatory and evoked SCR (Bach et al., 2010a; Staib et al., 2015) under a canonical response function (Bach et al., 2009, 2010b; Gerster et al., 2018). Specifically, a fixed-dispersion response at CS onset (with latency between 0 and 2 s) and a fixed-latency response at (potential) US onset were estimated for each trial. The inversion algorithm was not informed about trial type or the presence of an US. This method has been successfully used for quantifying threat memory in similar studies setups (Bach et al., 2010a, 2018a; Staib et al., 2015; Staib and Bach, 2018; Tzovara et al., 2018). We included only nonreinforced trials in the analysis to avoid any contamination by US responses.

##### HPR.

We detected R spikes in the ECG using a modified Pan–Tompkins algorithm implemented in PsPM (Paulus et al., 2016). Inter beat interval was mapped onto the time point of the following R spike, and values outside 400 and 1200 ms (corresponding to a heart rate between 50 and 150 bpm) excluded. Heart period was then linearly interpolated with 10 Hz sampling frequency and filtered with a fourth order bidirectional bandpass Butterworth filter (cutoff frequencies: 0.015–0.5 Hz). To estimate the anticipatory pupil response, we used a conditionwise general linear convolution model (Castegnetti et al., 2016).

#### Statistical analysis

Statistical analysis was done in R (www.r-project.org), version 3.3.1, using the R function aov() for ANOVAs and R package lme4, version 1.1.15, for linear mixed effects (LME) models together with package lmerTest for Sattertwaithe approximation to degrees of freedom (Luke, 2017). We analyzed trialwise response estimates (SEBR, PSR, SCR) in LME models. For PSR and SCR, only trials without US entered analysis. This model can deal with unbalanced data such that exclusion of individual trials is unproblematic. LME models included fixed effects for drug, CS, drug × CS, and for the effect of time in retention and relearning (trial number across CS for retention and within CS for relearning), as well as their interactions, together with a random intercept (R model formula: startle ∼ drug*CS*time, random = 1 |subject). Including other random effects rendered the models inestimable. Fixed effects statistics were extracted using the function anova(). Conditionwise heart period was tested in a standard repeated-measures ANOVA and fixed effects tested against pooled error variance. Control measures were tested for group differences with independent samples *t* tests without correction for multiple comparisons.

Cross-validation analysis of our main result was performed using a simplified ANOVA model that does not take into account the randomized trial sequence. We first replicated the main result using a drug × CS^+^/CS^−^ × time (trial number within CS) ANOVA, using the R package ezANOVA, version 4.4–0. We then predicted each participant's CS^+^/CS^−^ difference from the drug factor, in a threefold cross-validation scheme. We randomly partitioned our participant sample into 3 equally sized folds. Because the partitioning affects the results, the procedure was repeated on 10 random partitionings. We trained a linear model on two folds and predicted the CS^+^/CS^−^ difference in the third fold. Residual variance proportion was computed as sum of squared prediction error, divided by the number of data points, and by the variance of the data. We then randomly permuted participants' drug labels 1000 times and repeated the procedure. For each permutation, residual variance proportion was averaged over the 3 folds and the 10 partitionings. A *p-*value was computed as the rate by which the residual sum of squares in the random permutations was smaller than when using the correct drug labels.

#### Data and code availability

All anonymized data are available in a public repository (https://doi.org/10.5281/zenodo.3441715). All specific code used to generate the results and figures is available at https://doi.org/10.17605/OSF.IO/UJHXW.

## Results

### Acquisition of CS/US association before drug application

On acquisition visit 2, participants performed a discriminant delay threat conditioning task (Fig. 1*C*) in which two CS^+^ coterminated with an aversive electrical stimulation in 50% of trials, while a single CS^−^ was never reinforced. Accuracy in an incidental task (Table 1) was (nonsignificantly) higher for the doxycycline group and was subsequently modeled as a covariate to corroborate our primary analysis of memory retention.

**Figure 2.**
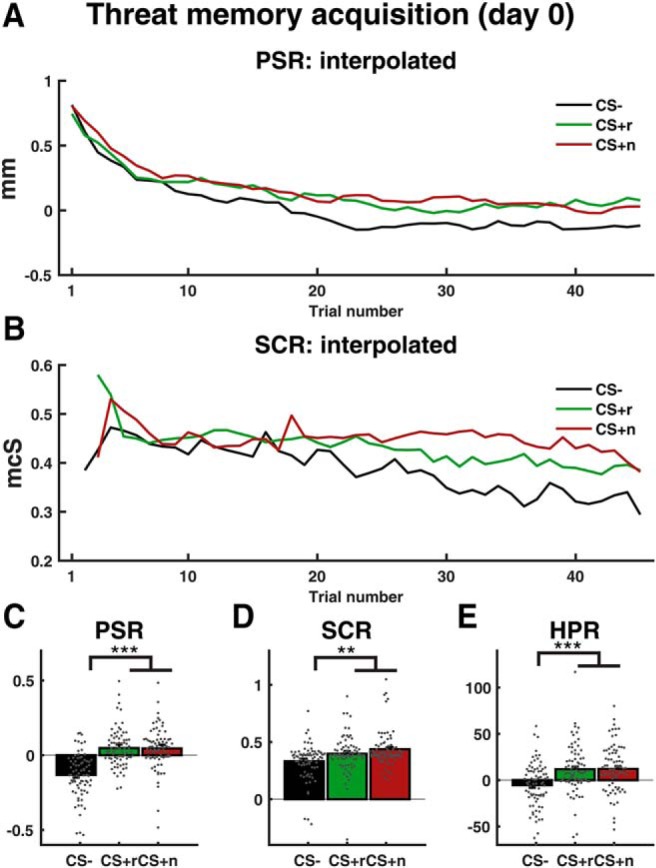
Learning indices during threat acquisition on day 0. ***A***, ***B***, Trial-by-trial PSR and SCR data, interpolated with last observation carried forward. ***C***, PSR last 15 trials interpolated and averaged. ***D***, SCR last 15 trials interpolated and averaged. ***E***, HPR across all trials. Error bars refer to between-subject SEM of conditionwise estimates after correcting for the overall participant mean. Scatter plots show individual participants' response, after correcting for the overall participant mean. **p < 0.01; ***p < 0.001 (Table 2).

Participants learned the CS/US association as indicated by stronger PSR, SCR, and HPR, to both CS^+^ than to CS^−^ (Fig. 2, Table 2). PSR (but not SCR or HPR) CS^+^/CS^−^ differences were higher for the placebo than for the doxycycline group. Also, PSR and SCR (but not HPR) to CSr^+^ were higher than to CSn^+^, although both CS^+^ had the same global reinforcement rate, and were randomized in terms of position in the trial sequence and local reinforcement rate.

**Table 2.** Linear mixed-effects models (trialwise PSR and SCR) and ANOVA (conditionwise HPR) results for the acquisition phase on day 0, i.e. 7 d before drug ingestion

	*F*	*df*	*p*
PSR: Group	0.49	1, 78.3	0.49
PSR: CS^+^ vs CS^−^	150.89	1, 3390.8	<0.001
PSR: Group × (CS^+^ vs CS^−^)	3.89	1, 3390.8	0.049
PSR: CSr^+^ vs CSn^+^	4.19	1, 2226.3	0.041
PSR: Group × (CSr^+^ vs CSn^+^)	1.1	1, 2226.3	0.29
PSR last 15 trials: CS^+^ vs CS^−^	90.99	1, 1101.6	<0.001
PSR last 15 trials: Group × (CS^+^ vs CS^−^)	2.68	1, 1101.6	0.1
PSR last 15 trials: CSr^+^ vs CSn^+^	0.06	1, 713.9	0.8
PSR last 15 trials: Group × (CSr^+^ vs CSn^+^)	0	1, 713.9	1
SCR: Drug	0.69	1, 76	0.41
SCR: CS^+^ vs CS^−^	15.39	1, 2182	<0.001
SCR: Group × (CS^+^ vs CS^−^)	0.26	1, 2182	0.61
SCR: CSr^+^ vs CSn^+^	6.97	1, 1012	0.008
SCR: Group × (CSr^+^ vs CSn^+^)	0.77	1, 1012	0.38
SCR last 15 trials: CS^+^ vs CS^−^	7.38	1, 669.1	0.007
SCR last 15 trials: Group × (CS^+^ vs CS^−^)	1.23	1, 669.1	0.27
SCR last 15 trials: CSr^+^ vs CSn^+^	0.05	1, 666.3	0.83
SCR last 15 trials: Group × (CSr^+^ vs CSn^+^)	0.26	1, 666.3	0.61
HPR: Group	1.54	1, 76	0.22
HPR: CS^+^ vs CS^−^	15.51	1, 154	<0.001
HPR: Group × (CS^+^ vs CS^−^)	0	1, 154	0.94
HPR: CSr^+^ vs CSn^+^	0	1, 76	0.95
HPR: Group × (CSr^+^ vs CSn^+^)	2.46	1, 76	0.12

However, analyzing just the final 15 trials of the acquisition session revealed a clear CS^+^/CS^−^ difference with no difference between the two CS^+^ (Table 2) and no difference between the two groups. Thus, we conclude that both CS^+^ were ultimately associated with US to the same extent in both experimental groups. To account for any possible differences between the groups, overall CS^+^/CS^−^ difference in PSR (across all trials) was subsequently modeled as a covariate to corroborate our primary analysis of memory retention.

### Increased CS^+^ retention 1 week after CSr^+^ reminder under doxycycline

**Figure 3.**
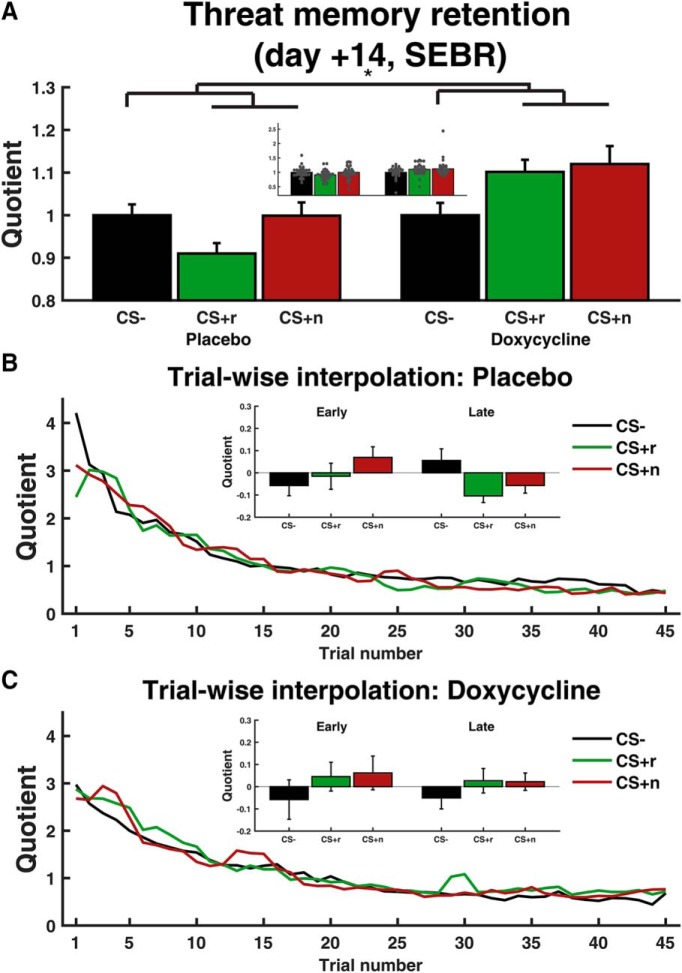
SEBR during threat memory retention on day +7. ***A***, Average over all trials. Inset, Same data overlaid with individual participants' responses, after correcting for the overall participant mean. ***B***, ***C***, Trial-by-trial data, interpolated with last observation carried forward. Insets, Early (first 15 trials) and late (last 15 trials). To account for the random trial sequence and therefore unbalanced distribution of data points across time, the insets show difference from an exponential habituation curve, fitted across all trials per participant. An LME with exponential habituation (instead of the omnibus effect of trial) yielded the same result pattern as shown in Table 3. Error bars refer to between-subject SEM of conditionwise estimates after correcting for the overall participant mean. *p < 0.05.

Seven days after acquisition visit 2, participants ingested placebo or 200 mg doxycycline. After 3.5 h they were exposed to an unreinforced CSr^+^. Then all electrodes were detached and they watched a 10 min cartoon movie, followed by a neuropsychological assessment. Seven days later (i.e., on day +14), we measured threat memory retention under extinction (i.e., with no US presentation) as our primary outcome (Fig. 3*A*, Table 3). Fear-potentiated startle was measured as SEBR to acoustic startle probes on each of 45 extinction trials, and analyzed in a LME model with trial number as predictor across CS types, to account for the individually randomized trial sequence.

**Table 3.** Linear mixed-effects models results for the retention test 7 d after drug ingestion/reminder and 14 d after acquisition

	*F*	*df*	*p*
SEBR: Drug	4.32	1, 87.7	0.041
SEBR: CS^+^ vs CS^−^	1.43	1, 3254.7	0.23
SEBR: Trial	33.45	44, 3264.8	<0.001
SEBR: Drug × (CS^+^ vs CS^−^)	4.39	1, 3254.7	0.036
SEBR: Drug × trial	0.87	44, 3264.8	0.72
SEBR: Trial × (CS^+^ vs CS^−^)	1.5	44, 3314.7	0.018
SEBR: Drug × trial × (CS^+^ vs CS^−^)	1.04	44, 3314.7	0.4
SEBR: CSr^+^ vs CSn^+^	1.06	1, 2086.6	0.3
SEBR: Drug × (CSr^+^ vs CSn^+^)	0.24	1, 2086.6	0.62
SEBR: Drug × trial × (CSr^+^ vs CSn^+^)	1.01	44, 2137.8	0.46
SEBR: CS^+^ vs CS^−^ (placebo)	0.41	1, 1671.6	0.52
SEBR: Trial (placebo)	19.51	44, 1677.2	<0.001
SEBR: Trial × (CS^+^ vs CS^−^) (placebo)	1.68	44, 1703.9	0.004
SEBR: CS^+^ vs CS^−^ (doxycycline)	5.37	1, 1583.2	0.021
SEBR: Trial (doxycycline)	14.8	44, 1587.1	<0.001
SEBR: Trial × (CS^+^ vs CS^−^) (doxycycline)	0.9	44, 1608.9	0.67

In the placebo group, we observed extinction learning (CS × trial interaction, see Fig. 3*B*, insets) and startle habituation (main effect trial). There was no difference between CSr^+^ and CSn^+^ in this group, or time × CSr^+^/CSn^+^ interaction, suggesting that the experimental procedure, which involved a 60 min neuropsychological test after the reminder, had no appreciable impact on differential reconsolidation. The doxcycline group showed no evidence for extinction learning and instead a persistent CS^+^/CS^−^ difference, again with no difference between CSr^+^ and CSn^+^ (for statistics, see Table 3).

Comparing the two groups in our primary analysis revealed in doxycycline-treated individuals a larger SEBR overall and in particular for CS^+^ (main effect drug, drug × CS^+^ interaction). This interaction was clearly visible on integrated EMG traces, suggesting that this difference is not due to any possible effects of doxycycline treatment on the timing or shape of the startle response which could bias its scoring. Across both groups, SEBR habituated (main effect trial), and the initially higher SEBR under CS^+^ relative to CS^−^ extinguished over time (interaction CS^+^ × trial). There was no overall difference between CSr^+^ and CSn^+^, and no impact of doxycycline on this difference. Because of evidence for differential learning in the two groups already on day 1 (as indexed by CS^+^/CS^−^ difference in PSR), we included this parameter into the model as a covariate. This replicated the drug × CS^+^ interaction and revealed no significant effect involving the covariate. The same result was observed in a model that included accuracy during initial learning as covariate. Thus, there was no evidence to suggest that our main result was better explained by group differences in initial learning or performance.

Because this significant result stands in contrast to our prior expectations, there is an increased risk that it represents a false positive and that indeed doxycycline has no systematic effect in the population. We therefore used cross-validation and investigated how well the observed drug × CS^+^ interaction generalized within the sample. To facilitate this analysis, we did not take into account the randomized trial sequence. We first replicated our main result in an drug × CS^+^/CS^−^ × trial (per CS) ANOVA (drug × CS^+^/CS^−^: *F*_(1,76)_ = 7.40, *p* = 0.008). Cross-validation analysis showed that a participant's CS^+^/CS^−^ difference could be predicted from whether a participant had taken drug or placebo, using a model that had not seen this participant's data (random permutation test: *p* < 0.001). This suggests that the observed drug × CS^+^/CS^−^ is consistent within our sample.

### Reduced CS^+^ relearning 1 week after CSr^+^ reminder under doxycycline

**Figure 4.**
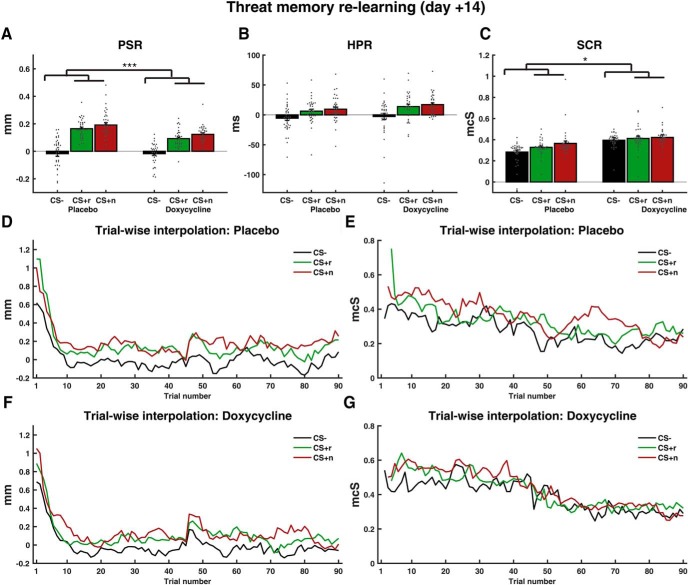
Threat memory measures during relearning on day +14. ***A*** and ***C***, Trialwise estimates averaged over all 90 trials. ***B***, Conditionwise estimates averaged across all 90 trials. ***D***–***G***, Trial-by-trial data interpolated with last observation carried forward. Trials for which <10 participants provided data (due to the random trial sequence) are not plotted. Reinforced trials were not analyzed; first trial was always reinforced. Error bars refer to between-subject SEM of conditionwise estimates after correcting for the overall participant mean. Scatter plots show individual participants' response after correcting for the overall participant mean. *p < 0.05, ***p < 0.001.

Next, we analyzed the relearning session, which immediately followed the retention session and always started with a reinforced CS^+^ trial (Fig. 4, Table 4). We observed larger PSR and SCR to CS^+^ versus CS^−^ in the placebo group than in the doxycycline group (interaction drug × CS) and no difference between, or interaction with, CSr^+^ and CSn^+^. SCR were overall higher after doxycycline than placebo treatment. There was no impact of drug on HPR. Across both groups, PSR, SCR and HPR were higher for CS^+^ than CS^−^. Initially high PSR and SCR decayed over time (main effect trial).

**Table 4.** Linear mixed-effects models (trialwise PSR and SCR) and ANOVA (conditionwise HPR) results for the relearning test 7 d after drug ingestion/reminder and 14 d after acquisition

	*F*	*df*	*p*
PSR: Drug	0.45	1, 71.5	0.51
PSR: CS^+^ vs CS^−^	316.17	1, 6279.2	<0.001
PSR: Trial	36.25	15, 6282.6	<0.001
PSR: Drug × (CS^+^ vs CS^−^)	19.46	1, 6279.2	<0.001
PSR: Drug × trial	1.45	15, 6282.6	0.12
PSR: Trial × (CS^+^ vs CS^−^)	0.98	14, 6281.4	0.47
PSR: Drug × trial × (CS^+^ vs CS^−^)	0.96	14, 6281.4	0.5
PSR: CSr^+^ vs CSn^+^	1.46	1, 4148.7	0.23
PSR: Drug × (CSr^+^ vs CSn^+^)	0.04	1, 4148.7	0.83
PSR: Trial × (CSr^+^ vs CSn^+^)	0.92	15, 4155.5	0.54
PSR: Drug × trial × (CSr^+^ vs CSn^+^)	1.18	15, 4155.5	0.28
SCR: Drug	4.03	1, 69	0.049
SCR: CS^+^ vs CS^−^	20.93	1, 4131	<0.001
SCR: Trial	23.04	14, 4131	<0.001
SCR: Drug × (CS^+^ vs CS^−^)	5.1	1, 4131	0.024
SCR: Drug × trial	1.33	14, 4131	0.18
SCR: Trial × (CS^+^ vs CS^−^)	0.81	14, 4131	0.66
SCR: Drug × trial × (CS^+^ vs CS^−^)	0.41	14, 4131	0.97
SCR: CSr^+^ vs CSn^+^	2.79	1, 2001	0.095
SCR: Drug × (CSr^+^ vs CSn^+^)	1.03	1, 2001	0.31
SCR: Trial × (CSr^+^ vs CSn^+^)	0.8	14, 2001	0.67
SCR: Drug × trial × (CSr^+^ vs CSn^+^)	0.78	14, 2001	0.69
HPR: Drug	0.39	1, 70	0.54
HPR: CS^+^ vs CS^−^	18.11	1, 142	<0.001
HPR: Drug × (CS^+^ vs CS^−^)	0.62	1, 142	0.43
HPR: CSr^+^ vs CSn^+^	0.63	1, 70	0.43
HPR: Drug × (CSr^+^ vs CSn^+^)	0	1, 70	0.95

Separating the groups, we observed higher PSR (*F*_(1,3223.2)_ = 235.2, *p* < 0.001) and SCR (*F*_(1,2064.0)_ = 28.5, *p* < 0.001) to CS^+^ versus CS^−^ in the placebo group, and higher PSR (*F*_(1,2875.9)_ = 116.8, *p* < 0.001) but not SCR (*F*_(1,1887.0)_ = 2.7, *p* = 0.10) to CS^+^ versus CS^−^ in the doxycycline group. There was no CS^+^/CS^−^ × trial interaction in the placebo group, which is expected given that the first available data point refers to a trial after at least one US.

## Discussion

In this study, we sought to demonstrate that the nonselective MMP inhibitor doxycycline disrupts threat memory reconsolidation, as a proof-of-principle for its clinical application. We based this hypothesis on the fact that many molecular and cellular features of consolidation and reconsolidation are shared, and on our previous observation that doxycycline disrupts threat memory acquisition/consolidation. However, contrary to our expectations, threat memory reminder under doxycycline had no specific impact on the reminded CS^+^. Instead, the manipulation appeared to globally increase CS^+^/CS^−^ discriminative memory during retention test, compared with placebo. This increased discriminative memory was consistent within our sample, as demonstrated using cross-validation. Tentatively, this may be due to reduced extinction learning during the retention test, in those individuals that were reminded under doxycycline, although a direct comparison of the extinction trajectory between the two groups was not significant. Furthermore, subsequent threat relearning was reduced in those that were reminded under doxycycline. Together, it appears that doxycycline may globally impair memory 1 week later. While unexpected, this result offers important insights into the potential role of MMPs in memory. We discuss possible scenarios that could explain our current and previous data (Bach et al., 2018a).

Explaining the lack of a reminder-specific effect of doxycycline in the present data (but not global memory impairment), there is a possibility that MMP-9 is involved in consolidation, explaining our previous result (Bach et al., 2018a), but not in reconsolidation. Despite the conceptual similarity of consolidation and reconsolidation (McKenzie and Eichenbaum, 2011) and overlap in the molecular pathways, important differences have also been pointed out (comprehensively reviewed in Besnard et al., 2012). For example, norepinephrine antagonists (McGaugh, 2000; Debiec and Ledoux, 2004; Lonergan et al., 2013) and gamma-aminobutyric acid agonists (Makkar et al., 2010) block both consolidation and reconsolidation. Also, translational control in mTOR signaling-dependent manner (Roesler, 2017), and transcriptional control through NF-κB downstream signaling (de la Fuente et al., 2015) appear involved in consolidation and reconsolidation. Conversely, an example for pathway dissociation is the involvement of brain-derived neurotrophic factor BDNF in consolidation but not reconsolidation, and of the transcription factor Zif268 in reconsolidation but not consolidation (Lee et al., 2004). Our data suggest that MMP-9 may be involved only in memory consolidation. In one rodent study, memory reconsolidation was attenuated by inhibiting MMP-9; however, that study did not support the otherwise well established effect of MMP-9 inhibition on synaptic consolidation such that this result offers ambiguous evidence (Brown et al., 2009). As a limitation, doxycycline is an unspecific MMP inhibitor. There is evidence that MMPs other than MMP-9 are involved in learning and memory (Meighan et al., 2006; Conant et al., 2015), although the underlying signaling pathways and proteolytic targets are less well known, for mainly methodological reasons (Huntley, 2012). In case diverse MMPs have different, possibly even opposing, roles for consolidation, and/or for reconsolidation, then unspecific MMP inhibition could reveal results that are difficult to interpret. Overall, it appears that more work is needed in nonhuman animals to establish the signaling pathway involved in memory consolidation, and the contribution of MMP-9. It has been suggested that an impact of MMP-9 on LTP involves its substrate CD44, a transmembrane protein and receptor for the ECM component hyaluron (Bijata et al., 2017). However, many other substrates of MMP-9 could potentially confer an impact on learning and memory as well. For example, dystroglycan, another transmembrane protein and part of ECM, has been reported as a MMP-9 substrate (Michaluk et al., 2007). Dystroglycan and dystrophin-dystroglycan complex are localized at hippocampal GABAergic synapses (Brünig et al., 2002). Cell-specific loss of dystroglycan from hippocampal pyramidal cells leads to distinct loss of GABAergic CCK positive basket cell terminals, with defect in hippocampal theta oscillations (Früh et al., 2016). Theta oscillations have been associated with memory function in both rodents and humans (Hebscher et al., 2019), including threat memory retrieval (Seidenbecher et al., 2003; Khemka et al., 2017a; Tzovara et al., 2019). Doxycycline inhibition of MMP-9 could thus interfere with GABAergic transmission and alter network oscillations that are integral to cognition and memory.

Regarding global memory impairment beyond the clearance of the drug (but not the lack of a reminder-specific effect), several explanations appear plausible. First, it is possible that MMP inhibition, and thus an impact of doxycycline on LTP, lasts for more than a week. Doxycyline is reported not only to inhibit MMP activity (Golub et al., 1991), but also MMP synthesis, reducing mRNA levels (Hanemaaijer et al., 1998). If doxycycline exerts this impact by blocking the ribosome, because ribosomal RNA has a turnaround time of >2 weeks (Mathis et al., 2017), it is possible that full level of MMP translation is not achieved 1 week after doxycycline ingestion, leading to lingering reduction in LTP. More tentatively, it is also possible that the effects of MMP on memory are not (only) conferred via LTP but via other mechanisms, including the configuration of extracellular matrix. Indeed, doxycycline affects extracellular matrix structure (Palomino-Morales et al., 2016), and different structural components of the matrix are suggested to impact on memory (Gogolla et al., 2009; Tsien, 2013; Happel et al., 2014; Banerjee et al., 2017). The turnaround time of the extracellular matrix is much longer than that of individual proteins (Tsien, 2013), thus explaining a long-lasting impact of doxycycline treatment. Finally, it is possible that doxycycline acts on memory via a pathway not involving MMP. For example, doxycycline induces apoptosis in cancer stem cells (Matsumoto et al., 2017) and may have the same impact on neuronal progenitor cells. This could explain an effect at least on hippocampal-dependent memory, which would last longer than 1 week since adult new born neurons require around 28 d to proliferate after acquiring the status of neuronal progenitor cells from stem cells, migrate to the granular zone from the sub granular zone and send out dendrites to integrate into the network (Abrous and Wojtowicz, 2015). We note that our human data cannot disambiguate these possibilities and further *in vitro* research will be required to answer this question.

As a limitation, our conclusion that doxycycline induces a lasting memory impairment is partly based on impaired extinction learning after doxycycline treatment. This however is a tentative interpretation of our data, based on demonstrating globally stronger discriminative memory retention in doxycycline-treated individuals, together with evidence for extinction learning during the retention test in placebo-treated individuals, and lack of such evidence in doxycycline-treated individuals. However, a direct statistical comparison of extinction learning between both groups was not significant, such that this should be investigated in a larger sample. Measuring at least serum concentration of doxycycline could also help account for behavioral variability and thus increase the sensitivity of the assessment.

Furthermore, the conclusion of a difference between doxycycline impact on consolidation and reconsolidation also merits replication. We note that demonstration of reconsolidation blockade in human threat conditioning has generally been more mixed than in nonhuman animals, both regarding behavioral (Kredlow et al., 2016) and pharmacological interventions (Elsey et al., 2018). This may be due to suboptimal experimental circumstances as well as to large interindividual variability. We note that our power calculations were based on the best-case assumption of negligible variability of the true drug effect and variability only in the measurement. In case of non-negligible or even high variability across individuals, much larger sample sizes may be required.

To summarize, we find no evidence of a specific impact of CS^+^ reminder under doxycycline on memory reconsolidation. Instead, we find a global impairment in extinction learning, and threat relearning, in doxycycline-treated individuals, which lasted beyond the clearance of the drug.
